# Low-Dose Blue Light (420 nm) Reduces Metabolic Activity and Inhibits Proliferation of Human Dermal Fibroblasts

**DOI:** 10.3390/life13020331

**Published:** 2023-01-25

**Authors:** Anne K. E. Brüning, Jennifer L. Schiefer, Paul C. Fuchs, Patrick Petzsch, Karl Köhrer, Christoph V. Suschek, Ewa K. Stürmer, Christian Opländer

**Affiliations:** 1 Clinic for Thoracic and Cardiovascular Surgery, Heart and Diabetes Center North Rhine-Westphalia, University Hospital, Ruhr-University Bochum, 32545 Bad Oeynhausen, Germany; 2 Plastic Surgery, Hand Surgery, Burn Center, Cologne-Merheim Hospital, Witten/Herdecke University, 51109 Cologne, Germany; 3 Genomics & Transcriptomics Laboratory, Heinrich Heine University Düsseldorf, 40225 Düsseldorf, Germany; 4 Department for Orthopedics and Trauma Surgery, Medical Faculty, Heinrich-Heine-University Düsseldorf, 40225 Düsseldorf, Germany; 5 Department of Vascular Medicine, University Heart Center, University Medical Center Hamburg-Eppendorf (UKE), 20251 Hamburg, Germany; 6 Institute for Research in Operative Medicine (IFOM), Cologne-Merheim Medical Center, Witten/Herdecke University, 51109 Cologne, Germany

**Keywords:** blue light, wound infection, fibrosis, proliferation, scarring, burns

## Abstract

Hypertrophic scarring in burn wounds is caused by overactive fibroblasts and myofibroblasts. Blue light reveals wavelength- and dose-dependent antibacterial and antiproliferative effects and may serve as a therapeutic option against wound infection and fibrotic conditions. Therefore, we evaluated in this study the effects of single and multiple irradiations with blue light at 420 nm (BL_420_) on the intracellular ATP concentration, and on the viability and proliferation of the human skin fibroblast (HDFs). In addition, possible BL_420_-induced effects on the catalase expression and differentiation were assessed by immunocytochemical staining and western blot analyses. Furthermore, we used RNA-seq analyses to identify BL_420_-affected genes. We found that BL_420_ induced toxicity in HDFs (up to 83%; 180 J/cm^2^). A low dose of 20 J/cm^2^ reduced the ATP concentration by ~50%. Multiple irradiations (4 × 20 J/cm^2^) inhibited proliferation without visible toxicity and reduced catalase protein expression by ~37% without affecting differentiation. The expression of about 300 genes was significantly altered. Many downregulated genes have functions in cell division/mitosis. BL_420_ can strongly influence the fibroblast physiology and has potential in wound therapy. However, it is important to consider the possible toxic and antiproliferative effects, which could potentially lead to impaired wound healing and reduced scar breaking strength.

## 1. Introduction

The human skin is challenged every day by environmental influences. In particular, sunlight has a profound impact on the skin physiology. Here, solar UV-B (280–315 nm) and UV-A (315–400 nm) exerts many biological and mutagenic effects, causing sun burns, skin cancer, and premature skin ageing [1,2]. On the other hand, UV-radiation can also have a positive impact on the human physiology, for example, the regulation of the body homeostasis via the activation of the central neuroendocrine system and UV-B mediated production of vitamin D [3,4]. Apart from UV radiation, the spectrum of terrestrial solar radiation reaching the earth’s surface also comprises visible light (50%) and infrared radiation (45%) [5].

Many previous studies have shown that blue light (400–470 nm) has antimicrobial properties against many microorganisms and also clinically relevant pathogens, such as gram-positive and gram-negative bacteria, mycobacteria, molds, yeasts, and dermatophytes [6]. Thus, blue light is often used in the treatment of acne vulgaris [7]. However, due to its relatively high energy, blue light (400–500 nm) has toxic effects on the human eye, inducing photochemical injury to the retina, called photoretinitis [8].

Some studies have shown that blue light inhibits the proliferation of many cells types, such as pig kidney embryo cells [9], gingival fibroblasts [10], human keratinocytes [11] and dermal fibroblasts in vitro [12]. Further studies showed that the irradiation with blue light (470 nm) inhibits the growth of skin tumors in mice and improves the wound healing in rats [13,14]. Here, an induction of the angiogenesis and improvement of ischemic wound healing were observed [15]. Moreover, blue light treatment has anti-inflammatory properties, can reduce psoriatic plaques observed in psoriasis patients, and also has positive effects on severe atopic dermatitis without the depletion of Langerhans cells and T-cells in the skin often observed during UV-treatment [16,17,18].

Dermal fibroblasts synthesize and organize the extracellular matrix (ECM), maintaining the mechanical properties of the skin [19]. In the wound healing process, dermal fibroblasts release the necessary cytokines and growth factors, and differentiate partly into myofibroblasts that mediate the wound granulation, wound contraction, and reconstruction of skin structures [20,21].

The UVA penetrates more deeply into the skin and dermis, which in turn can have negative effects on dermal fibroblasts by inducing intracellular stress resulting in the deterioration of the dermal extracellular matrix, followed by the loss of skin elasticity and wrinkling [22]. It is thought that blue light can penetrate even deeper than UVA, reaching the lower dermis and subdermis [23].

We previously demonstrated that blue light can induce toxicity in dermal fibroblasts in vitro, dependent on the wavelength and dose. Particularly, irradiation with LED arrays with shorter wavelengths within the violet spectrum (410, 420 nm) showed pronounced toxicity, whereas when using longer wavelengths (>453 nm), no toxic effects could be observed.

We found lower proliferation rates using blue light (410, 420, 453 nm) irradiation with non-toxic doses, with the exception of blue light at 480 nm [24]. A recent study confirmed the potential toxic and antiproliferative effects of blue light at 420 nm, depending on cell types and light doses [25].

It is supposed that photons interact with endogenous/intracellular molecules acting as photoreceptors. Thus, blue light has effects on cytochrome C oxidase and lipofuscin, inducing the generation of singlet oxygen and/or other reactive oxygen species (ROS), which in turn may be responsible for some blue light-induced effects [26,27,28].

Furthermore, flavoproteins and flavoenzymes using FAD and/or FMN as cofactors may act as flavin-based photosensors and be affected by blue light [29].

It has been described, that in humans, 90 genes with flavin-dependent proteins mainly involved in primary metabolic pathways, such as the citric acid cycle, β-oxidation, and degradation of amino acids, used either FMN dermal (16%) or FAD (84%) [30]. Other flavoproteins, such as NADPH oxidase and nitric oxide synthase, use NADPH as a cofactor [31], producing high amounts of ROS or reactive nitrogen species under proinflammatory conditions.

Recently, we found that non-toxic blue light (453 nm) irradiations in higher doses (80 J/cm^2^) inhibited the proliferation and myofibrogenesis of foreskin fibroblasts, which was accompanied by a decrease in the intracellular FAD concentration and also a decrease in the NADP^+^/NADPH ratio, indicating that FAD/flavoproteins can undergo photoreduction [32]. In addition, although blue light irradiations did not show toxicity, a decay of the catalase expression and a fast increase in intracellular ROS were observed.

On one hand, the modulation of skin cell proliferation and differentiation could be a therapeutic tool for some pathological skin conditions, such as hypertrophic scarring after burns and scleroderma. On the other hand, it is very likely that, besides UV radiation, blue light also plays a certain role in dermal photoaging and skin cancers and may hamper the wound healing process.

Since there are a lot of open questions related to the blue light-induced effects of blue light and underlying mechanisms, we have investigated the general impact of single and repeated light irradiations of low-dose blue (420 nm) on the cell metabolism, viability, proliferation, differentiation, and gene transcription of human dermal fibroblasts.

## 2. Materials and Methods

### 2.1. Materials

If not otherwise indicated, chemicals were purchased from Sigma-Aldrich (Munich, Germany) and the cell culture media and supplements were purchased from PAN Biotech (Aidenbach, Germany).

### 2.2. Skin Specimen

The skin samples for cell isolation were donated from six female patients (27–46 years old, mean 36.5 years) undergoing reduction abdominoplasty (5×) or upper tight lift (1×) with the donor consent and the vote of approval of the Ethics Commission of Witten/Herdecke University (ID 15/2018), and in accordance with the Declaration of Helsinki.

### 2.3. Cell Culture

After surgery, skin specimens were transported in a sterile container on ice. In the laboratory, the skin specimens were disinfected with ethanol (70%, 1 min) and washed (3×) with phosphate-buffered saline (PBS, 1% penicillin/streptomycin). Single skin samples were taken using biopsy punches (8 mm; Biopsy Punch, Servoprax GmbH, Wesel, Germany). Each skin punch sample was freed of the fatty/connective tissue using scissors and forceps and placed in a well of a cell culture plate (12-well, Sarstedt, Nümbrecht, Germany) with 700 µL of the cell culture media (DMEM, w/o phenol red, 10% fetal bovine serum, 1% penicillin/streptomycin, 1% l-Glutamine). Here, the dermis had contact with the bottom of the cell culture. After incubation (7 d; 37 °C; 5% CO_2_), the skin punch samples were carefully lifted, and the migration of fibroblasts was microscopically investigated. The adherent fibroblasts were further cultivated and cryoconserved as described elsewhere [24]. Prior to the cell culture experiments, the cryoconserved stocks of fibroblasts were thawed and cultured (5% CO_2_, 37 °C) in T 75 cell culture flasks (Sarstedt, Nümbrecht, Germany). One day before the experiments, the cells were washed by three rinses with PBS and detached by an incubation with 2.5 mL TrypLE Express solution (Gibco, Thermo Fisher Scientific, Denmark) for 4 min. The enzyme activity was neutralized by 10 mL of DMEM/FBS. After the centrifugation (5 min/400× *g*) and resuspension, fibroblasts were counted using an automated cell counter (Nucleocounter NC-100, Chemometec, Allerod, Dänemark) and seeded in cell culture dishes (35 mm, Greiner Bio-One GmbH, Frickenhauser, Germany). For the western blot experiments, the positive control TGF-β (10 ng/mL Peprotech, Hamburg, Germany) for the induction of myofibroblast differentiation was added from the d3. For all cell experiments, the used fibroblasts were in lower passages <7.

### 2.4. Irradiation of Human Dermal Fibroblasts

A LED Light Bar (420 nm Violet Spectrum, BML Horticulture, Austin, TX, USA) with a wavelength of 420 +/− 11 nm was adjusted to deliver 33.6 mW/cm^2^ (±1.5%) using a calibrated densiometer (RM21; Dr. Gröbel, Ettlingen, Germany).

For the determination of toxic effects, six days after seeding the 1.5 × 10^4^ cells/culture dish (35 mm; 9.6 cm^2^) fibroblasts were irradiated in parallel for 0, 5, 10, 20, 30, or 60 min; therefore, with a dose of 0, 10, 20, 40, 60, or 120 J/cm^2^, in an open cabinet. Directly before the irradiation, the cell culture media in the dishes were replaced by 1 mL PBS. After irradiation, the irradiated dishes were kept in another open cabinet with the unirradiated control until the 60 min irradiation ended. Then, the PBS was replaced by the fresh cell culture media and the fibroblast cultures were placed in the incubator (37 °C; 5% CO_2_). The evaporation was diminished by covering the dishes with transparent lids (absorbance < 5%). During the irradiation, the dishes were cooled by ventilation. Under these conditions, the temperature in the irradiated dishes and buffers never exceeded 34 °C, whereas without cooling, temperatures above 41 °C were reached.

For the low-dose irradiation (10 min; 20 J/cm^2^), the cells were treated the same way, with the exception that PBS was replaced in the control (0 J/cm^2^) and irradiated dishes directly after irradiation by the fresh cell culture media. Dishes were placed in the incubator as fast as possible. For ATP measurements, 2.25 × 10^4^ cells/culture dish (35 mm) were used.

### 2.5. Determination of Cell Viability, Proliferation, and Cell Toxic Effects

Possible cell detachments after irradiation were excluded by microscopic examination using a Leica light microscope (Leica, Wetzlar, Germany). The fibroblast viability in relation to the untreated control was determined by a resazurin-based assay (CellTiter-Blue^®^, Promega, Walldorf, Germany). Cells were incubated with the CellTiter-Blue^®^ reagent (1 h; 1:10 with medium; 1000 µL/well) and samples of supernatants (3 × 100 µL) were measured using a spectrometer (Epoch II, BioTek, Winooski, VT, USA) at wavelengths 573 nm and 605 nm. In parallel, cell deaths were investigated microscopically using a fluorescence microscope (DMI4000B, Leica, Wetzlar, Germany) and the fluorescence dyes fluorescein diacetate (0.5 µg/mL) and Hoechst 33342 (1.0 µg/mL). In each experiment, three different images/cell culture wells were taken, and the cell number and the percentage of living and dead cells were analyzed and evaluated by the ImageJ^®^ software (v. 1.53 k) [33].

### 2.6. Myofibroblast Differentiation

For visualizing the impact of blue light on myofibroblast differentiation on day 7 and day 10 after seeding, fibroblasts were fixed by a 15 min treatment with 4% paraformaldehyde/PBS and permeabilized by 0.2% Triton X-100/PBS. Cells were incubated for 30 min with blocking buffer (4% BSA/PBS), followed by a 60 min incubation (37 °C) with mouse anti-α-SMA-antibodies (αSMA, ab7817, Abcam, Cambridge, UK) in blocking buffer (1:400). Cells were washed three times with PBS and incubated for 60 min with an AlexaFluor488-conjugated goat-anti-mouse antibody (Invitrogen, Carlsbad, CA, USA) in the blocking buffer (1:1000). After three washing steps with PBS, cells were incubated with Hoechst 33342 (1.0 µg/mL) in PBS for 10 min. After one washing step, the cells were visualized by fluorescence microscopy. The numbers of nuclei in a field of view at 100× magnification (4 fields of view/well) were evaluated by the ImageJ^®^ software.

### 2.7. Determination of ATP

ATP measurements in fibroblasts were conducted 1 h after irradiation. Prior to the ATP determination, the medium of fibroblast cultures were removed and cells were washed with 1 mL PBS. After, the addition of 200 µL PBS/well cells were detached using a cell scraper (Greiner Bio-One GmbH, Frickenhausen, Germany, and transferred to 1.5 mL centrifugation tubes (Sarstedt, Nümbrecht, Germany)). The cell suspensions were centrifuged (17,000× *g*, 10 min), the supernatants were discarded, and the cell pellets were resuspended in 250 µL of boiling Milly-Q water and mixed by 3 × 5 s vortexing, which led to the breakdown of the cell membrane [34]. Between vortexing, the samples were kept in a heat block (100 °C) and were mixed with a 1000 µ pipette (Eppendorf, Hamburg, Germany). The mixtures were centrifuged again (17,000× *g*, 5 min), and each supernatant was transferred to a fresh 1.5 mL centrifugation tube and stored at −80 °C until the ATP measurements using a luminescence-based ATP determination kit (Biaffin, Kassel, Germany). For the measurements, stock solutions and ATP standards (0–8 μM) were prepared as described in the manufacturer’s protocol, and the samples (50 µL) and prepared reaction mix (50 μL) were mixed in a white 96-well microtiter plate. The chemiluminescence of samples was measured after an exposure time of 20 min at a wavelength of 590 nm using a fluorescence spectrometer (VICTOR II, Perkin Elmer, Waltham, MA, USA). The amounts of ATP were calculated based on the standard and normalized to the number of cells.

### 2.8. Western Blotting

At the time points indicated (24 h after treatment), four fibroblast cultures in cell culture plates (35 mm) of each single patient were washed 3 times with 4 °C cold PBS, and 50 µL RIPA-lysis buffer/14.2% protease inhibitor (cOMPLETE, Roche, Sigma-Aldrich, Munich, Germany) was added onto the cells. The cell suspensions were collected, pooled, and frozen at −80 °C for 1 h and then thawed at room temperature for 32 min. After mixing by a pipette the cell lysates, the supernatants were centrifuged for 10 min at 15.000× *g*/4 °C and transferred to fresh precooled tubes, and cryoconserved at −80 °C.

Prior to the western blotting using the XcellSureLock Mini-Cell-System (Invitrogen, Karlsruhe, Germany) under reducing conditions, the samples were thawed, and the protein concentrations were determined using the Pierce™ BCA Protein Assay Kit (#23225, Thermo Fisher, Dreieich, Germany) according to the manufacturer´s protocol. The samples (30 µg protein each) were loaded onto the SDS-PAGE (TGX Stain-Free Gel, Biorad, Feldkirchen, Germany). After electrophoresis, gels were visualized by Imager, ChemiDoc™XRS (BioRad, Hercules, CA, USA) and the total amount of protein was quantified the Quantity One 1-D Analysis Software Version 4.6.5 (BioRad). Blotting was performed using nitrocellulose membrane (Peqlab, Erlangen, Germany) and the Trans-Blot^®^ Turbo™ Transfer System (Bio Rad, CA, USA). The successful protein transfer was verified via Ponceau red S staining. The obtained nitrocellulose membranes were incubated (16 h) in T-TBS (5% non-fat milk, 0.1% Tween 20) at 4 °C. After the incubation with primary antibodies (anti-catalase mouse monoclonal antibody, clone OTI1B8; OriGene, Rockville, USA) or with a monoclonal mouse anti-human α-smooth muscle actin (αSMA, ab7817, Abcam, Cambridge, UK) according to the manufacturer’s instructions, membranes were washed (3 × 5 min in T-TBS) and incubated (1 h) with goat anti-mouse IgG (DAKO, Glostrup, Denmark). After further washing steps (4 × 3 min, T-TBS), the Clarity ^TM^ Western ECL Substrate (BioRad/#170-5060) was used, and the signals of bound antibodies were detected and analyzed.

### 2.9. RNA-Seq Analyses

In parallel to the proliferation assays, at days 7 and 10 (24 h after treatment), RNA samples were prepared from two fibroblast well culture dishes (35 mm) using the RNeasy Mini Kit (Qiagen, Hilden, Germany) according to the manufacturer`s protocol. The obtained total RNA samples were quality measured by capillary electrophoresis using the Fragment Analyzer and the ‘Total RNA Standard Sensitivity Assay’ (Agilent Technologies Inc., Santa Clara, CA, USA), and quantified for transcriptome analyses (Qubit RNA HS Assay, Thermo Fisher Scientific). All samples in this study have very high RNA quality (Quality Numbers, RQN; mean = 10.0). According to the manufacturer’s protocol using the ‘VAHTS™ Stranded mRNA-Seq Library Prep Kit’ for Illumina^®^ library, preparations were performed. Briefly, the total RNA (300 ng) was used for mRNA capturing, fragmentation, the synthesis of cDNA, adapter ligation, and library amplification. Bead purified libraries were normalized and finally sequenced (read setup 1 × 150 bp) on the HiSeq 3000/4000 system (Illumina Inc., San Diego, CA, USA). We used the bcl2fastq tool to convert the bcl files to fastq files, as well as for adapter trimming and demultiplexing. Data analyses on fastq files were conducted using CLC Genomics Workbench (versions 12.0.3 and 20.0.2, QIAGEN, Venlo. NL). The reads of all probes were adapter trimmed (Illumina TruSeq) and quality trimmed (default parameters: bases below Q13 were trimmed from the end of the reads, ambiguous nucleotides maximal 2). Mapping was performed against the *Homo sapiens* (hg38) (25 May 2017) genome sequence. After the grouping of samples (for biological replicates each) according to their respective experimental condition, multi-group comparisons were made and statistically determined using the empirical analysis of DGE (version 1.1, cutoff = 5). The resulting *p* values were corrected for multiple testing by FDR and Bonferroni correction. A *p* value of ≤0.05 was considered significant. All results of fibroblasts isolated from upper tight were excluded from the comparisons because of their different origin of skin area, which led to considerable differences already in the controls when compared to the fibroblasts isolated from the abdominoplasties.

### 2.10. Statistical Analysis

Except for the RNA-seq analyses, the results of all cell experiments were statistically analyzed by the GraphPad Prism Version 8.4.3 (San Diego, CA, USA). Significant differences were evaluated using one-way ANOVA. A *p*-value of <0.05 was considered significant.

## 3. Results

### 3.1. Blue Light Effects on Cell Proliferation and Cell Viability

**Figure 1:**
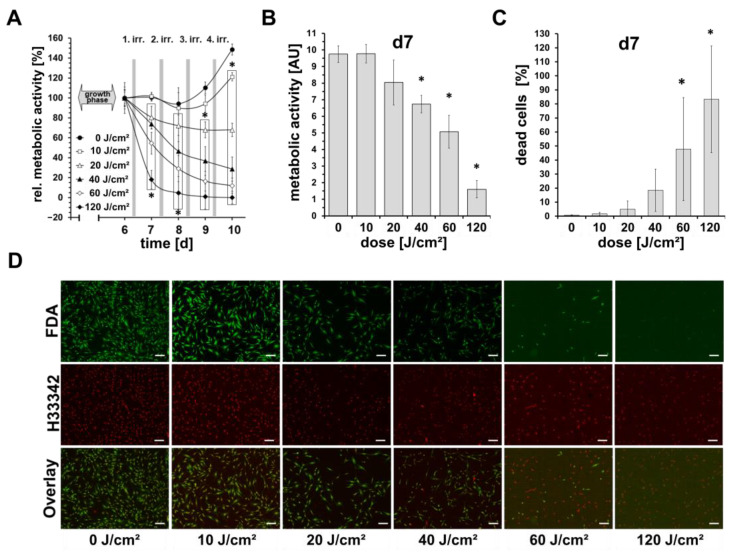
Effects of blue light on cell viability and proliferation. Shown are mean ± SD values of 5 independent experiments, * *p* < 0.05 as compared to the control values. (**A**) Dermal fibroblasts were irradiated daily with different doses (0–120 J/cm^2^) of blue light (420 nm; 33.6 mW/cm^2^) on 4 consecutive days. Cell viability was determined by a resazurin-based assay on time points, as indicated and normalized to the individual initial resazurin values on day 6 before irradiations. (**B**) Shows raw resazurin values after the first single blue light irradiation measured (16 h after irradiation). (**C**) Parallel quantitative determination of dead/live cells ratios obtained from live cell imaging using Hoechst 33342- and fluorescein diacetate (FDA) staining on day 7. (**D**) Shown are representative microphotographs of Hoechst 33342, and FDA-stained fibroblast cultures on day 7, 24 h after irradiation. Viable cells show a green fluorescence signal of cytoplasma by FDA. Nuclei were stained by Hoechst 33342 and the signals were colored in red for better visualization. Bars = 200 µm.

In screening experiments, the proliferation and viability of blue light-irradiated fibroblasts were measured from day 6 until day 10 (4 irradiations in total) every 24 h, using a resazurin-based assay. As shown in Figure 1A, the normalized signal of the unirradiated control fibroblasts on days 7 and 8 was similar to that on day 6. Only from day 9 onwards an increase in the signal by 10% can be seen, and on day 10 by 50%. In comparison, at the highest dose used (daily 120 J/cm^2^), there we observed a strong reduction in the cell viability. Here, on day 7 after one irradiation, the viability signal was below 20%, and from day 8 almost at 0%.

Daily irradiations with 40 or 60 J/cm^2^ also showed a significant reduction, but to a lesser extent; on day 6, the signal was about 70% and 55%, respectively, and on day 10, somewhat at 30% and 10%, respectively. Irradiations with 20 J/cm^2^ showed only a slight reduction by a maximum of 25%, while irradiations with 10 J/cm^2^, after an initial reduction by about 15% until day 9, showed an increase in the signal above the initial level on day 10 (105%).

Figure 1B shows the results of (not normalized) cell viability/metabolic activity assessed by resazurin-based assays 16 h after the first irradiation. Here, a significant dose-dependent decrease in the resazurin signal can be observed, which shows a good inverse correlation to the relative number of dead cells assessed by the live-cell imaging as shown in Figure 1C,D. Only for doses of 10 J/cm^2^ and 20 J/cm^2^, no significant differences to the control could be observed, neither in the resazurin-assays nor in the live-cell imaging.

### 3.2. Low-Dose Blue Light Effects on Intracellular ATP Concentration and Cell Proliferation

**Figure 2:**
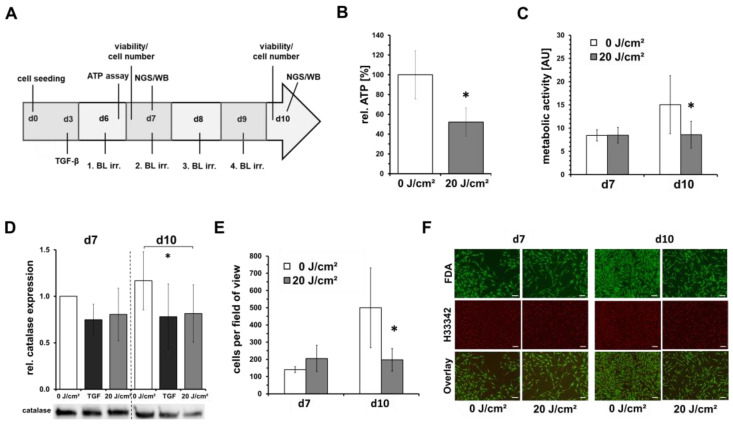
Low-dose blue light irradiations inhibit fibroblast proliferation. Shown are mean ± SD values of 5 independent experiments, * *p* < 0.05 as compared to the control values. Human dermal fibroblasts were irradiated with low, sub-toxic doses (20 J/cm^2^) of blue light (420 nm). (**A**) Flowchart of experimental procedure. Human dermal fibroblasts were seeded on day 0 for experiments and grown for 6 days. On d6–d9, fibroblast cultures were irradiated by blue light (BL; 420 nm; 33.6 mW/cm^2^) and cell culture media was changed on a daily basis. Determinations of cell viability, cell number, intracellular ATP concentration, sample preparation for next generation sequencing (NGS) and Western blot (WB) were performed as indicated. As positive control, TGF-β (10 ng/mL) was added for some experiments. (**B**) Results of intracellular ATP-concentration 1 h after irradiation on day 6. (**C**) Metabolic activity assessed by a resazurin-based assay and (**D**) relative catalase protein expression assessed by western blot 16 h or 24 h, respectively, after one irradiation on day 7 and four irradiations on day 10. (**E**) Quantitative determination of dead/live cell ratios obtained from live cell imaging using Hoechst 33342- and fluorescein diacetate (FDA) staining. (**F**) Shown are representative microphotographs of Hoechst 33342- and FDA-stained fibroblast cultures (50×). Viable cells show a green fluorescence signal of cytoplasma by FDA. Nuclei were stained by Hoechst 33342, and the signals were colored in red for better visualization. Bars = 200 µm.

Having shown that blue light irradiation at doses of 10 and 20 J/cm^2^ has no significant toxic effect on fibroblasts, we chose a low-dose of 20 J/cm^2^ for the further experiments to investigate the possible inhibition of the proliferation. The experimental schedule is shown in Figure 2A. Preliminary studies have shown that blue light (453 nm) at higher doses can significantly decrease intracellular ATP concentration in irradiated fibroblasts. Therefore, the effect of a single, low-dose irradiation (20 J/cm^2^) with blue light, but here with a wavelength of 420 nm, on the intracellular ATP concentration of fibroblasts, was investigated. It was found that shortly after irradiation (1 h), the intracellular ATP concentration in irradiated cells was approximately halved compared to the unirradiated control (Figure 2B). In this experimental set-up, we also evaluated the metabolic activity/viability by resazurin-based assay on day 7 (1 × irradiation) and day 10 (4 × irradiations), and observed a significant reduction in the resazurin signal of about 30% for the irradiated cells on day 10 compared with untreated cells (Figure 2C).

Furthermore, by Western blotting, a significant reduction in the catalase protein expression was observed on day 10, thus, after 4 blue light irradiations (Figure 2D). Live-cell-imaging using Hoechst 33342 and FDA did not show any blue light-induced toxicity but counting Hoechst 33342-positive nuclei showed a significant cell number reduction of about 60% (Figure 2E,F).

### 3.3. Low-Dose Blue Light Did Not Induce Fibroblast/Myofibroblast Differentiation

**Figure 3:**
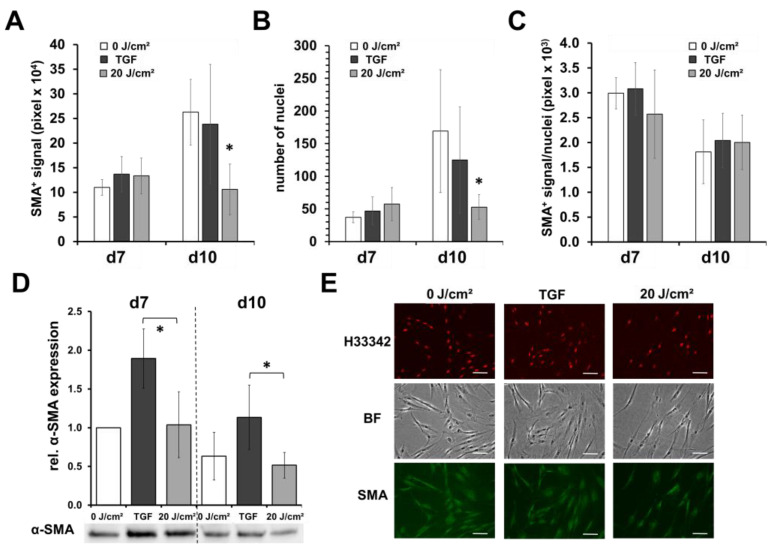
Blue light does not induce myofibroblast differentiation. Shown are the means ± SD of 5 experiments with different patients (* *p* < 0.05). Human dermal fibroblasts were irradiated with low, sub-toxic doses (20 J/cm^2^) of blue light (420 nm). On day 7 (24 h after 1st blue light irradiation) and on day 10 (4× blue light irradiations), fibroblasts were fixed, subsequently immunocytochemically stained with antibody against α-smooth muscle actin (α-SMA), a myofibroblast marker, and evaluated by fluorescence microscopy. As positive control, TGF-β (10 ng/mL; TGF) was added from d3. (**A**) Determination of the pixel number of the positive α-SMA fluorescence signal or (**B**) number of nuclei (Hoechst 33342^+^) in a field of view at 100× magnification (4 fields of view/well). (**C**) Ratio of α-SMA/number of nuclei. (**D**) Relative protein expression of α-SMA assessed by Western blot analysis. (**E**) Shown are representative microphotographs (200×) of α-SMA- and Hoechst 33342-stained fibroblast cultures on day 10 (bright field; BF). Hoechst 3342 signals were colored in red for better visualization. White bars = 50 µm.

To exclude a possible differentiation of fibroblasts, which among other things is associated with a reduced cell number at relatively high metabolic rates, we examined the expression of intracellular alpha smooth muscle actin (SMA) as a marker of myofibroblasts in blue light-irradiated and non-irradiated fibroblasts by Western blot and immunocytochemical staining. As shown in Figure 3A,E, on day 10, the total signal of SMA by digital evaluation of fluorescence microscopy photographs of stained fibroblast cultures was significantly reduced by about 58–62%, in comparison with the unirradiated controls or fibroblasts supplemented with TGF-β as the positive control. However, the number of cells was also significantly reduced (68% vs. control; 58% vs. TGF-β supplemented fibroblasts), as shown by nuclei-counting (Figure 3B); thus, after the normalization of the SMA signal to the number of cells (nuclei), no significant differences were observed between the treatments (Figure 3C).

Nevertheless, as shown by Western blot analysis, the intracellular amount of SMA (normalized to cell protein content) was significantly increased by TGF-β supplementation (Figure 3D). Here, between the irradiation and non-treated controls, no significant differences could be noticed; thus, an increased induction of myofibroblast could be denied.

### 3.4. Effects of Low-Dose Blue Light Irradiation on Gene Expression of Human Skin Fibroblasts

Blue light can have a wide variety of effects on the cell biology depending on the wavelength, dose, irradiance, and irradiation time. Not much is known about the effect of blue light on fibroblasts at 420 nm in low, sub-toxic doses; therefore, we performed next-generation sequencing (NGS) to analyze the gene expression to obtain the possible evidence of blue light-induced mechanisms and to identify any genes/proteins affected.

As pictured in the experimental schedule (see Figure 2A), we examined the transcription of fibroblasts by NGS on day 7, therefore, 24 h after irradiation. Furthermore, further analyzes of gene expression were performed on day 10, after a total of four daily irradiations and 24 h after the last irradiation. After grouping the samples according to the respective experimental conditions, multi-group comparisons were performed and statistically analyzed. The resulting 𝑃 values were corrected for multiple testing using the FDR and Bonferroni correction. Only gene expression changes with a factor ≥2 were also considered. A summary of the gene expression results is shown in Table 1.

It was found that on day 7, a single blue light irradiation resulted in a relatively lower number of significant gene expression changes compared with day 10 after repeated irradiations (e.g., compare and Con d7 vs. BL d7 with Con d10 vs. BL d10). The most changes in the gene expression with respect to the respective unirradiated cells were seen by blue light after four irradiations on day 10 (Con (d10) vs. BL (d10)). After the Bonferroni correction here, the expression of around 300 different genes was affected by blue light. However, it was also shown that, during the culture period, the expression pattern of the fibroblasts changed; for example, after the Bonferroni correction there were expression changes of 129 genes in the unirradiated controls (see Con d7 vs. Con d10).

As can be seen in Table 1 (see Con d7 vs. BL d10), a single blue light irradiation causes changes in the gene expression of two genes (≥2×; Bonferroni correction), namely Sequestosome-1 (SQSTM1) and Leucine-rich repeat containing protein 32 (LRRC32).

Multiple (4×) blue light irradiations on a daily basis changed the gene expression of fibroblasts. After the Bonferroni correction, the expression of 296 genes was significantly altered (see Table 1: Con d10 vs. BL d10). Of these, upregulation was observed for 102 genes and down-regulation for 194 genes. For overview purposes, Table 2 lists upregulated genes whose expression change factor was greater than or equal to four.

Table 3 lists some genes that showed downregulation of the gene expression after multiple blue light irradiations (factor ≥ 4). The gene expression of acyl-CoA binding domain containing 7 (ACBD7) and leiomodin 1 (LMOD1) is reduced the most by about a factor of 10. In addition, many genes involved in the cell cycle or mitosis are down-regulated, for example, Serine/threonine-protein kinase Nek2 (NEK2), Kinesin family member 20A (KIF20A), Suppressor APC domain-containing protein 2 (SAPCD2), Cell division cycle protein 20 homolog (CDC20), Protein FAM83D (FAM83D), Serine/threonine-protein kinase, PLK1(PLK1), Histone H3-like centromeric protein A (CENPA), G2/mitotic-specific cyclin-B1 (CCNB1), and G2/mitotic-specific cyclin-B2 (CCNB2).

## 4. Discussion

During wound healing, fibroblast proliferation and myoblast differentiation are crucial for the reestablishment of the extracellular matrix and barrier function of the skin by secreting, for example, extracellular proteins, such as collagen, and growth factors [21,35]. Nevertheless, in fibrotic conditions such as Dupuytren’s disease or excessive scarring after burns, deregulated myofibroblasts are considered to be the effector cells responsible for scarring, contraction, and excessive collagen production [36,37].

Blue light can affect skin cell physiology similarly to UV radiation, and depending on the dose, intensity, wavelengths, and frequency of exposure, blue light irradiation can be potentially harmful but also has medical therapeutic uses [38].

In this study, we were able to confirm previous results showing that blue light at a wavelength of 420 nm induced toxic effects on human skin fibroblasts [24]. Our recent results show that cell toxicity was up to 82% (60 min; 120 J/cm^2^), but also we could demonstrate that low doses ≤20 J/cm^2^ did not show toxic effects at the first sight. However, multiple blue light irradiations with low doses (20 J/cm^2^) led to a significant reduction in the cell number, which was accompanied by a loss in the signal of alpha smooth muscle positive fibroblasts/myofibroblasts. Thus, as shown in Figure 3C, the fibroblast/myofibroblast ratio was not influenced by blue light, nor was the intracellular relative content of alpha smooth muscle protein (Figure 3D). In previous studies, we could show that low-dose blue light can inhibit or reduce the TGF-induced differentiation of fibroblasts into myofibroblasts, which was not the case in the present study. It seems that blue light is able to modify the TGF-β pathway, or activation. Without the endogenous addition of TGF-β, as in the present study, the blue light-induced inhibitory effects on the basic fibroblast/myofibroblast differentiation seem limited, and the inhibitory effects on proliferation come more into play. We could demonstrate that a single irradiation with 20 J/cm^2^ led to a fast and significant reduction of intracellular ATP concentration by ~50% (Figure 2B). Thus, a large part of the available chemical energy of the cells, which is required for metabolism division, proliferation, and differentiation, among other things, is no longer available after a blue light irradiation. The obtained results of this study support this assumption.

Multiple blue light irradiations reduced the metabolism (Figure 2C), cell number, and nuclei (Figure 2E and Figure 3E), and achieved protein concentrations (not shown) for the Western blot by about half.

The depletion of the intracellular ATP concentration by more than 50% after blue light irradiation in fibroblasts was also shown with blue light with a different wavelength (453 nm), but at a much higher dose (80 J/cm^2^) [32]. This indicates that blue light, dependent on the wavelength and dose, may cause mitochondrial dysfunction, possibly by the interaction of blue light with endogenous chromophores, for example, flavin-containing proteins and cytochromes with specific absorbance spectra, resulting in the intracellular generation of ROS, which in turn may hamper the respiration chain and therefore ATP generation [39,40,41].

In addition, blue light is also known to be able to make skin fibroblasts more sensitive to oxidative noxious agents such as hydrogen peroxide [24]. In the present study, low doses of blue light (420 nm; 20 J/cm^2^) were able to weaken the enzymatic antioxidative defense system of cells by reducing the concentration of cellular catalase, which neutralizes the detrimental effects of H_2_O_2_ [42]. Moreover, a photoinactivation of catalase induced by VIS and blue light may be possible, as described by Cheng et al. 40 years ago [43].

Therefore, we can assume that interfering with cell metabolism and weakening the cellular antioxidant system plays major roles in mediating the blue light-induced effects on human fibroblasts. By RNA-seq analyses, we observed that the transcriptional profile of many genes can be affected by single/multiple blue light treatments, as shown in Table 1.

Since the number of genes involved is high, it is not possible to consider each one and discuss possible functions within the scope of this work. In the following, the most important changes in the gene expression were looked at in terms of their factors and possible involvement in proliferation, differentiation, and the stress response to fibroblasts. 

To reduce the probability of false positives from multiple testing, the data were corrected according to Bonferroni. Here, it was shown that, especially the expression of two genes for SQSTM1 and LRRC32, were significantly upregulated even after single irradiation, as well as after multiple irradiations.

SQSTM1 encodes an adaptor protein (SQSTM1/p62) that is mainly involved in the transport, degradation, and destruction of various (misfolded/damaged) protein aggregates and cooperates with components of autophagy and the ubiquitin-proteasome degradation pathway [44]. The SQSTM1 promoter contains an antioxidant response element (ARE) and can be upregulated by oxidative stress via the transcription factor NF-E2-related factor 2 (Nrf2). In addition, oxidative stress-induced NFkB phosphorylation was shown to upregulate SQSTM1, resulting in an increase in the pigmented epithelial cell survival via increased autophagy activity [45]. The observed increased upregulation at the transcriptional level on days 7 and 10 could therefore primarily indicate blue light-induced oxidative stress.

LRRC32 is a key regulator of TGF-β and controls TGF-β activation. It encodes the protein glycoprotein-A repetitions predominant (GARP), a type I transmembrane cell surface docking receptor for latent transforming growth factor-β (TGF-β), which is abundantly expressed on regulatory T lymphocytes and platelets (ENSG00000137507-LRRC32). Here, GARP bound to the inactive pro-protein of TGF-β (proTGFβ) increases their enzymatic cleavage by furin-like proteases under the formation of latent TGF-β, hence soluble TGF-β molecules to which latency-associated peptides (LAP) are still bound. To form biologically active TGF-β, the LAP is removed via integrins αVβ6 and αVβ8 via protease-dependent and protease-independent mechanisms [46,47]. There are few studies investigating the function of GARP in human fibroblasts. Thus, the expression of GARP in fibroblasts has been demonstrated, but the activation of latent TGF-β has not been detected [48].

The strongest upregulation after multiple irradiations showed ketimine reductase mu-crystallin (117×). According to the Human Protein Atlas (ENSG00000103316-CRYM), this oxidoreductase with its ligands NAD and NADP is detected in many tissues, particularly in brain and heart tissues and also in skin. However, it seems that in the skin, protein expression is probably limited to melanocytes. It can probably regulate the free intracellular concentration of triiodothyronine by binding thyroid hormones [49].

Fibroblasts typically can enzymatically degrade various collagens via matrix metallopeptidases (MMPs) in their role in the organization of the dermal extracellular matrix [50]. A blue light-induced upregulation of MMP1 (15×) and MMP15 (4.8×) gene expression may be medically useful, for example, for the induction of the degradation of scar tissue/fibrosis. Thus, further investigations of the expression/activity of these MMPs after blue light treatment could be very interesting for the evaluation of the anti-fibrotic potential of blue light.

Growth differentiation factor 15 (GDF15) gene expression was also significantly increased. GDF15 is involved in the cellular stress response program and often is elevated after hypoxia, inflammation, and oxidative stress [51]. Interestingly, GDF15 has been used as a biomarker for primary mitochondrial diseases [52]. One could speculate that the increased expression could be caused by blue light-induced mitochondrial dysfunction. Therefore, further studies would be necessary to elucidate the role of GDF15 in the context of blue light-induced effects.

As listed in Table 3, the gene expressions of the acyl-CoA binding domain containing 7 (ACBD7) and leiomodin 1 (LMOD1) are most reduced by a factor of about 10.

As its name suggests, ACBD7 is responsible for binding acyl-CoA esters and is more generally involved in metabolism and acyl-CoA biosynthesis [53]. In addition, ACBD7 is involved in organelle contacts in the cytosol and may be important for lipid metabolism and organelle-linkage budding from the endoplasmic reticulum [54]. Thus, a reduction in ACBD7 by blue light could possibly result in deficiencies in metabolism and organelle formation, which, in turn, can also be associated with a reduced proliferation rate.

LMOD1, on the other hand, is commonly found in fibroblasts and is involved in the organization of the extracellular matrix. Here, it mediates the enucleation of actin filaments, the initial step in the formation of actin filaments from actin monomers in the course of polymerization on actin [55]. The blue light-induced reduction in actin filament formation may have significant effects on cell stability and motility, cell division, and on the formation of contractile structures, for example, in the course of myofibroblast differentiation, the formation of alpha smooth muscle actin [56]. Therefore, LMOD1 would be a promising candidate for further experiments and studies, as here its downregulation would affect proliferation and differentiation.

GAS2L3 (Growth arrest specific 2 like 3), which is a cytoskeletal linker protein possibly involved in the stabilization and formation of the actin/microtubullin network, is also markedly downregulated after blue light irradiation (−4.87×) [57].

Furthermore, many genes for proteins/enzymes involved in the cell cycle or mitosis are downregulated, e.g., NEK2, KIF20A, SAPCD2, CDC20, FAM83D, PLK1, CENPA, CCNB1, and CCNB2. Here, it is not clear whether there is a common mechanism of regulation, e.g., whether ATP deficiency is sufficient to induce a similar gene expression change, or whether specific structures, for example, transcription factors or signaling pathways, are directly affected by blue light. Nevertheless, we assumed that downregulation of these genes generally indicates reduced cell division/proliferation. In addition, many genes involved in the formation of contractile filaments were downregulated by blue light, which could be considered a cause or consequence of blue light-inhibited myofibroblast differentiation.

The expectation that the increased formation of intracellular ROS, which can be induced by blue light, also affects or increases the gene expression of antioxidant enzymes, such as superoxide dismutase, glutathione peroxidase, or catalase, could not be confirmed. Contrary to these expectations, blue light decreased the expression of catalase and even downregulated it at the protein level.

In vivo, the situation is more complicated, as other factors can affect the fibroblast in and around the wound area. Resident and immune cells produce many signaling molecules and can be affected by wounding, inflammation, and blue light exposure [16,58]. For example, in a recent study, Magni et al. could show that blue light (410–420 nm; 20.6 J/cm^2^, 0.69 W/cm^2^) induces a quicker healing process in a mouse model. The authors suggested that the photobiomodulation of the wounds with blue light evokes a mast cell response, which, in turn, stimulates an early inflammatory response, angiogenesis, and myofibroblast differentiation [59]. In patients (*n* = 12) with systemic sclerosis skin ulcers, the weekly treatment of the ulcers with blue light (400–430 nm, 120 mW/cm^2^, 7.2 J/cm^2^), in addition to the standard therapy, showed significant improvements after 8 weeks [60]. The same device was used in a study for the treatment of chronic wounds. Here, blue light treatment, in addition to standard care, accelerates the re-epithelialization rate of chronic wounds [61].

Furthermore, the release of neurotransmitters, cytokines, and other factors by nerve endings in the skin plays an important role in the stress response to the skin [62]. Blue light irradiation may also have effects on nerve endings and the release of factors, which, in turn, may influence the physiology and behavior of fibroblasts. Thus, although blue light may inhibit or reduce fibroblast/myofibroblast activity, the wound healing process is not necessarily inhibited by blue light irradiation, at least at low doses. On the contrary, low-dose blue light therapy seems to stimulate and accelerate wound healing in vivo.

In summary, this work could show that especially short wavelength blue light can strongly influence cell physiology even at low doses. Besides the inhibition of proliferation and the reduction in the intracellular ATP concentration by blue light, many effects on various genes are observed at the transcriptional level, of which an involvement in the photobiological processes was unknown until now. Thus, this work was able to identify some interesting effects and candidate genes and forms an important basis for further studies.

## 5. Conclusions

Blue light irradiation has a certain medical potential in wound therapy, in particular against excessive scarring and wound infections. Nevertheless, it is important to be concerned about the possible toxic and antiproliferative effects, and to weigh up the use of blue light well with regard to a risk-benefit profile in order to minimize the disturbances of the skin physiology, which, in turn, may lead to impaired wound healing/closure, and reduced scar breaking strength.

## Figures and Tables

**Table 1:** Overview results of RNA-seq analyses. Shown are significant changes of gene expression of human fibroblasts (↑upregulation; ↓downregulation). Comparisons were performed of non-irradiated cells (con) and blue light (BL)-irradiated cells (20 J/cm^2^) 24 h after treatment on day 7 and of non-irradiated/irradiated fibroblasts on day 10, with a total of four daily irradiations and 24 h after the last irradiation (*n* = 4).

Comparison	Genes	Up and Down	ncRNA	Genes	Up and Down	ncRNA	Genes	Up and Down	ncRNA
con (d7) vs. BL (d7)	2	↑ 2	0	6	↑ 4	0	321	↑ 170	16
		↓ 0	0		↓ 2	0		↓ 151	8
con (d10) vs. BL (d10)	296	↑ 104	2	703	↑ 268	15	1426	↑ 601	51
		↓ 192	1		↓ 435	8		↓ 825	40
con (d7) vs. con (d10)	129	↑ 72	0	351	↑ 218	6	1077	↑ 736	60
		↓ 57	1		↓ 133	3		↓ 341	19
BL (d7) vs. BL (d10)	84	↑ 29	1	269	↑ 114	10	869	↑ 486	64
		↓ 55	0		↓ 155	2		↓ 383	20
	Bonferoni: *p* ≤ 0.05 |FC|: ≥2			FDR: *p* ≤ 0.05 |FC|: ≥2			*p*-value: *p* ≤ 0.05 |FC|: ≥2		

**Table 2:** Blue light-induced upregulation of gene expression in human fibroblasts. Shown are significant upregulated genes (≥4×) assessed in blue light-irradiated (20 J/cm^2^) and non-irradiated human fibroblasts on day 10, with a total of four daily irradiations and 24 h after the last irradiation (*n* = 4).

Database Object Name	ID	Fold-Change
Ketimine reductase mu-crystallin	CRYM	117.32
AC004988.1	*Antisense*	20.77
Interstitial collagenase	MMP1	15.04
FAM65C	*lincRNA*	14.97
Ras-related GTP-binding protein D	RRAGD	13.05
Growth/differentiation factor 15	GDF15	12.46
N-acylglucosamine 2-epimerase	RENBP	10.64
Proepiregulin	EREG	10.24
Integrin beta-3	ITGB3	9.80
Folate receptor gamma	FOLR3	8.65
Hexokinase-2	HK2	6.78
FAM87B	*lincRNA*	6.45
Sodium/potassium-transporting ATPase subunit beta-1-interacting protein 1	NKAIN1	6.29
Paraneoplastic antigen-like protein 6A	PNMA6A	5.87
Neuronal pentraxin receptor	NPTXR	5.34
Transmembrane glycoprotein NMB	GPNMB	4.87
Matrix metalloproteinase-15	MMP15	4.87
17-beta-hydroxysteroid dehydrogenase 14	HSD17B14	4.78
Dipeptidyl peptidase 4	DPP4	4.74
Leucine-rich repeat-containing protein 32	LRRC32	4.63
Pleckstrin homology-like domain family A member 1	PHLDA1	4.41
Uridine diphosphate glucose pyrophosphatase	NUDT14	4.13
Sequestosome-1	SQSTM1	4.03
cGMP-dependent protein kinase 2	PRKG2	4.03
F-box only protein 32	FBXO32	4.00

**Table 3:** Blue light-induced downregulation of gene expression in human fibroblasts. Shown are significant downregulated genes (≥4×) assessed in blue light-irradiated (20 J/cm^2^) and non-irradiated human fibroblasts on day 10, with a total of four daily irradiations and 24 h after the last irradiation (*n* = 4).

Database Object Name	ID	Fold-Change
Acyl-CoA-binding domain-containing protein 7	ACBD7	−15.59
Leiomodin-1	LMOD1	−10.89
Kinesin-like protein KIF20A	KIF20A	−7.23
RP11-867G23.10	*processed_transcript*	−6.55
SDPR	*protein_coding*	−6.53
Serine/threonine-protein kinase Nek2	NEK2	−6.50
PICALM interacting mitotic regulator	FAM64A	−6.37
Suppressor APC domain-containing protein 2	SAPCD2	−6.04
Alpha-N-acetylgalactosaminide alpha-2,6-sialyltransferase 5	ST6GALNAC5	−6.03
Cell division cycle protein 20 homolog	CDC20	−5.99
Protein FAM83D	FAM83D	−5.99
Oxytocin receptor	OXTR	−5.94
Tastin	TROAP	−5.67
Serine/threonine-protein kinase PLK1	PLK1	−5.51
Histone H3-like centromeric protein A	CENPA	−5.48
Cyclin-dependent kinase inhibitor 3	CDKN3	−5.37
Tumor necrosis factor alpha-induced protein 8-like protein 1	TNFAIP8L1	−5.29
Disks large-associated protein 5	DLGAP5	−5.25
G2/mitotic-specific cyclin-B1	CCNB1	−5.23
G2/mitotic-specific cyclin-B2	CCNB2	−5.22
Borealin	CDCA8	−5.14
Ras GTPase-activating-like protein IQGAP3	IQGAP3	−5.09
Centrosomal protein of 55 kDa	CEP55	−4.99
Lamin-B1	LMNB1	−4.98
GAS2-like protein 3	GAS2L3	−4.87
Abnormal spindle-like microcephaly-associated protein	ASPM	−4.78
Anillin	ANLN	−4.68
DEP domain-containing protein 1A	DEPDC1	−4.68
High mobility group protein B2	HMGB2	−4.67
Mitotic checkpoint serine/threonine-protein kinase BUB1	BUB1	−4.49
Cyclin-A2	CCNA2	−4.48
Protein regulator of cytokinesis 1	PRC1	−4.44
Matrilin-2	MATN2	−4.42
Protein Mis18-beta	OIP5	−4.32
Kinesin-like protein KIF20B	KIF20B	−4.30
Cyclin-F	CCNF	−4.29
RING finger protein 150	RNF150	−4.27
Kinesin-like protein KIF2C	KIF2C	−4.27
Kinetochore scaffold 1	KNL1	−4.25
Proline/serine-rich coiled-coil protein 1	PSRC1	−4.20
Baculoviral IAP repeat-containing protein 5	BIRC5	−4.16
RP11-265N7.1	*lincRNA*	−4.14
Proline-rich protein 15	PRR15	−4.13
Hyaluronan mediated motility receptor	HMMR	−4.12
Targeting protein for Xklp2	TPX2	−4.11
Securin	PTTG1	−4.09
Cell division cycle-associated protein 3	CDCA3	−4.08
Nucleolar and spindle-associated protein 1	NUSAP1	−4.05

## Data Availability

The data that supports the findings of this study are available from the corresponding author upon reasonable request.
